# Disrupted Bone Remodeling Leads to Cochlear Overgrowth and Hearing Loss in a Mouse Model of Fibrous Dysplasia

**DOI:** 10.1371/journal.pone.0094989

**Published:** 2014-05-01

**Authors:** Omar Akil, Faith Hall-Glenn, Jolie Chang, Alfred Li, Wenhan Chang, Lawrence R. Lustig, Tamara Alliston, Edward C. Hsiao

**Affiliations:** 1 Department of Otolaryngology, Head & Neck Surgery, University of California San Francisco, San Francisco, California, United States of America; 2 Department of Orthopaedic Surgery, University of California San Francisco, San Francisco, California, United States of America; 3 Endocrine Unit and Bone Imaging Core, San Francisco VA Medical Center, San Francisco, California, United States of America; 4 Division of Endocrinology and Metabolism, and the Institute for Human Genetics, Department of Medicine, University of California San Francisco, San Francisco, California, United States of America; Faculté de médecine de Nantes, France

## Abstract

Normal hearing requires exquisite cooperation between bony and sensorineural structures within the cochlea. For example, the inner ear secretes proteins such as osteoprotegrin (OPG) that can prevent cochlear bone remodeling. Accordingly, diseases that affect bone regulation can also result in hearing loss. Patients with fibrous dysplasia develop trabecular bone overgrowth resulting in hearing loss if the lesions affect the temporal bones. Unfortunately, the mechanisms responsible for this hearing loss, which could be sensorineural and/or conductive, remain unclear. In this study, we used a unique transgenic mouse model of increased G_s_ G-protein coupled receptor (GPCR) signaling induced by expression of an engineered receptor, Rs1, in osteoblastic cells. These ColI(2.3)^+^/Rs1^+^ mice showed dramatic bone lesions that histologically and radiologically resembled fibrous dysplasia. We found that ColI(2.3)^+^/Rs1^+^ mice showed progressive and severe conductive hearing loss. Ossicular chain impingement increased with the size and number of dysplastic lesions. While sensorineural structures were unaffected, ColI(2.3)^+^/Rs1^+^ cochleae had abnormally high osteoclast activity, together with elevated tartrate resistant acid phosphatase (TRAP) activity and receptor activator of nuclear factor kappa-B ligand (*Rankl*) mRNA expression. ColI(2.3)^+^/Rs1^+^ cochleae also showed decreased expression of Sclerostin (*Sost*), an antagonist of the *Wnt* signaling pathway that normally increases bone formation. The osteocyte canalicular networks of ColI(2.3)^+^/Rs1^+^ cochleae were disrupted and showed abnormal osteocyte morphology. The osteocytes in the ColI(2.3)^+^/Rs1^+^ cochleae showed increased expression of matrix metalloproteinase 13 (*MMP-13*) and *TRAP*, both of which can support osteocyte-mediated peri-lacunar remodeling. Thus, while the ossicular chain impingement is sufficient to account for the progressive hearing loss in fibrous dysplasia, the deregulation of bone remodeling extends to the cochlea as well. Our findings suggest that factors regulating bone remodeling, including peri-lacunar remodeling by osteocytes, may be useful targets for treating the bony overgrowths and hearing changes of fibrous dysplasia and other bony pathologies.

## Background

Bone remodeling, a critical process in the maintenance of skeletal homeostasis, integrates bone formation and bone resorption [1]. Abnormalities in bone remodeling cause significant morbidity, including deformity and disability associated with inherited diseases of abnormal bone formation and bone fragility associated with aging and osteoporosis. Not surprisingly, diseases that severely change bone remodeling can also affect hearing since the auditory transduction mechanism is embedded within bone [2]–[6].

Interactions between the structures of the cochlea, such as the bony otic capsule and the organ of Corti, are essential for hearing. In the embryo, the cochleae form through bidirectional signaling between the sensorineural structures and developing bone [7]. In adults, cells within the organ of Corti normally secrete soluble factors to suppress remodeling of cochlear bone by osteoclasts and osteoblasts [8], [9]. The importance of this crosstalk is evident in bone syndromes where these pathways are disrupted. For example, otosclerosis and osteogenesis imperfecta tarda are characterized by sensorineural, conductive, and mixed forms of hearing loss [10]–[12].

Patients with fibrous dysplasia (FD; OMIM #174800) of bone have lesions containing dense fibro-cellular infiltrate and increased trabecular bone formation. FD is caused by activating mutations in the G_s_ G-protein coupled receptor (GPCR) signaling pathway, which increases cyclic adenosine monophosphate (cAMP) levels [13]–[15]. As many as 20% of FD patients have hearing loss [16]–[22]. Disrupted bone remodeling leading to the overgrowth of temporal bones is thought to contribute to this progressive hearing loss in FD patients, which also can be either conductive, sensorineural, or both [18], [19]. However, the traditional methods of discriminating conductive *vs.* sensorineural hearing loss require intact bone physical properties to make that distinction. Thus our goal was to elucidate the mechanisms responsible for hearing loss in fibrous dysplasia.

In this study, we used a transgenic mouse model of increased G_s_-GPCR signaling to better define the mechanisms by which FD causes hearing loss. The complexity of the *GNAS* locus and embryonic lethality of constitutively-active G_s_α signaling [23] preclude direct genetic modifications to introduce the classical *GNAS* mutations that cause fibrous dysplasia. Since GPCRs signal through a limited number of canonical pathways including G_s_ and G_i_ that ultimately regulate intracellular cAMP levels, we developed a method of inducing regulated G_s_ signaling in cells by engineered receptors such as RASSLs (receptors activated solely by synthetic ligands). RASSLs are powerful tools for studying GPCR signaling as they no longer respond to endogenous hormones but can be activated by synthetic small-molecule ligands [24]. In addition, RASSLs are small genes easily expressed in constructs and transgenes allowing precise spatial and temporal regulation. RASSLs have proven to be useful for dissecting GPCR signaling in complex tissues including bone, brain, and heart [24]–[27].

Mice expressing the engineered G_s_-GPCR Rs1 in osteoblastic cells using the Collagen I 2.3 kb promoter fragment [ColI(2.3)^+^/Rs1^+^ mice] develop bone phenotypes that strongly resemble fibrous dysplasia of the bone, including increased trabecular bone formation, cortical erosions, and disorganized bone formation with rapid turnover and remodeling [26]. We used this dramatic phenotype to determine how FD-like lesions may affect the otic capsule and cochlea and elucidate the mechanisms that contribute to FD-induced hearing loss.

## Materials and Methods

### Mouse Strains

ColI(2.3)^+^/Rs1^+^ mice were generated as previously described [26] by mating mice with a collagen type 1α 2.3 kb promoter fragment driving the tTA (“TEToff” system) driver transgene [FVB/NJ-Tg(Col1a1-tTA)139Niss/Mmucd; MMRRC accession 030758-MU] with mice carrying the TetO-Rs1 responder transgene [FVB/N-Tg(tetO-HTR4*D100A)2Niss/Mmmh; MMRRC accession 029993-MU]. Double-transgenic mice [abbreviated here as ColI(2.3)^+^/Rs1^+^ mice] maintained off of doxycycline showed strong activation of G_s_-GPCR signaling in osteoblastic cells [26] and developed their fibrous dysplastic bone phenotype postnatally [28]. These mice were maintained on an FVBN background, a strain with minimal auditory defects [29]. Wild type [WT; ColI(2.3)^−^/Rs1^−^] or single-transgenic [ColI(2.3)^+^/Rs1^−^ and ColI(2.3)^−^/Rs1^+^] mice showed no identifiable bone phenotype and were collectively used as controls. Prior studies showed no sexual dimorphism in the bone phenotype [26], [28], [30] including differences in body length or weight. Our study combined the results from male and female mice for the morphological analyses and auditory testing. Only male mice were used for the gene expression and immunohistochemistry studies to minimize any potential effects of sex on bone formation and remodeling. This study was carried out in strict accordance with the recommendations in the Guide for the Care and Use of Laboratory Animals of the National Institutes of Health. All procedures and protocols were approved by the University of California, San Francisco Institutional Animal Care and Use Committee (#AN086974 and #AN098643). All procedures were performed under anesthesia, and all efforts were made to minimize suffering.

### Cochlear Morphology Assessments

A rank-order grading system was used to rate the condition and the severity of ColI(2.3)^+^/Rs1^+^ cochlear bone lesions based on the amount of bone overgrowth at 12 weeks observed by a single experimenter who was blinded to the hearing results at the time of scoring. Control cochleae categorized as a “cochlea with no abnormal bone overgrowth,” and correlated with evoked acoustic brainstem response (ABR) thresholds of 30 to 35 dB. Mild bony lesions were grouped based on the presence of small cochlear lesions, whereas moderate bony lesions were categorized as large growths on the cochlea and the labyrinth area. Severe bony lesions were grouped based on very large bone overgrowths involving the bulla and overgrowth on the labyrinth area.

### Auditory Assessments

Hearing tests were performed on control and ColI(2.3)^+^/Rs1^+^ littermates at 6 and 10–12 weeks of age in a soundproof chamber as described [31]–[33]. Briefly, mice were anesthetized by intraperitoneal injection of a mixture of ketamine hydrochloride (Ketaset, 100 mg/kg) and xylazine hydrochloride (Xyla-ject, 10 mg/kg). Body temperature was maintained with a heating pad and monitored throughout the hearing testing.

The evoked ABR thresholds were differentially recorded from the scalp of control and ColI(2.3)^+^/Rs1^+^ mice using subdermal needle electrodes at the vertex, below the pinna of the left ear (reference probe), and below the contralateral ear (ground probe). The sound stimuli included clicks (5 ms duration; 31 Hz) and tone pips at 8, 16, and 32 kHz (10 ms duration; cos2 shaping; 21 Hz). Measurements were recorded using the TDT BioSig III system (Tucker Davis Technologies). For each stimulus, electroencephalographic (EEG) activity was recorded for 20 ms at a sampling rate of 25 kHz and filtered (0.3–3 kHz). Waveforms from 512 stimuli were averaged for click responses, and 1000 stimuli for frequency specific stimuli (8, 16, and 32 kHz). ABR waveforms were recorded in 5 dB sound pressure level (SPL) intervals down from the maximum amplitude. The threshold was defined as the lowest stimulus level at which response peaks for waves I-V were clearly and repetitively present upon visual inspection. These threshold judgments were confirmed by analysis of stored waveforms. One-way ANOVA with Bonferroni post-hoc testing was used to determine statistical significance, defined as p<0.05.

Distortion product oto-acoustic emissions (DPOAE) were measured using an acoustic probe placed in the left external auditory canal. Stimuli consisted of two primary tones delivered simultaneously with a frequency ratio of f1/f2 = 1.25. Tones were digitally synthesized at 100 kHz using SigGen software. The primary tones with geometric mean (GM) frequencies ranging from 6 to 36 kHz and equal levels (L1 = L2 = 60 dB SPL) were presented *via* two separate speakers (EC1; Tucker Davis Technologies) to the acoustic probe. DPOAE responses (2f1–f2) were recorded using an ER10B microphone assembly (Etymotics Research) within the acoustic probe and the TDT BioSig III system (Tucker Davis Technologies). Responses were amplified, digitally sampled at 100 kHz, and averaged over 50 discrete spectra. Fast Fourier transforms were computed from averaged responses. For each stimulus set, the DPOAE amplitude level at 2f1–f2 was extracted, and sound pressure levels for data points 100 Hz above and below the DPOAE frequency were averaged for the noise floor measurements. DPOAE levels were plotted as a function of primary tone GM frequency. Statistical analysis was performed using ANOVA with Bonferroni post-hoc tests with significance defined as p<0.05.

### Histological Analysis

Histological analyses were performed as previously described for plastic [31]–[33] and paraffin embedded sections [34]. To histologically preserve the sensorineural structures in plastic sections, freshly-dissected cochleae were perfused through the round and oval windows with a solution of 2.5% paraformaldehyde and 1.5% glutaraldehyde in 0.1 M phosphate buffered solution (PBS) at pH 7.4. Cochleae were incubated in the same fixative overnight at 4°C, rinsed with 0.1 M PBS, and post-fixed in 1% osmium tetroxide for two hours prior to embedding in Araldite 502 resin (Electron Microscopy Sciences). 5 µm sections were stained with toluidine blue for histological analysis.

For paraffin sections, dissected cochleae were fixed in 4% paraformaldehyde in PBS overnight. Cochleae were decalcified by incubation at 4°C in 10% ethylenediaminetetraacetic acid (EDTA) for 2–4 days, followed by serial ethanol dehydration and embedding in paraffin. 6 µm thick sections were permeabilized in 0.3% Triton X-100 in PBS, processed for antigen retrieval with Ficin (Invitrogen), and blocked for intrinsic peroxidase activity with 3% hydrogen peroxide. Sections were rinsed in PBS/0.2% Tween (PBST) and blocked with 10% normal horse serum (Vector labs) for 2 h at room temperature. Sections were then incubated with the following primary antibodies overnight at 4°C: anti-MMP-13 (1∶50 dilution; Abcam, ab-39012) and anti-Sclerostin (1∶50 dilution; R&D systems, AF1589). Sections were rinsed with PBST and incubated with biotinylated secondary anti-goat and -rabbit antibodies (Vector labs), followed by PBST rinses and incubation with the ABC Elite avidin/biotin blocking kit (Vector labs). 3, 3′-diaminobenzidine (DAB) substrate peroxidase substrate antigen labeling kit (Vector labs) was used to visualize protein expression. Tartrate resistant acid phosphatase (TRAP) staining to assess osteoclast and osteocyte mediated bone remodeling in WT and FD cochleae was performed using the TRAP stained system (Sigma) as previously described [34].

### Whole-mount Immunofluorescence Hair Cell Analysis

Immunofluorescence studies were conducted on whole-mount cochleae of the ColI(2.3)^+^/Rs1^+^ and control mice as described [32], [33]. Cochleae were perfused with 4% PFA in 0.1 M PBS at pH 7.4 and kept in the fixative overnight at 4°C. Cochleae were then decalcified with 5% EDTA in 0.1 M PBS for 2–3 days. Following decalcification, the otic capsule and outer membranes were removed. The remaining organ of Corti was incubated with the anti-myosin VIIa antibody (a hair-cell specific marker; 1∶50 dilution in PBS; Proteus Biosciences, Cat# 25-6790) and incubated overnight at 4°C. Whole-mount cochleae were rinsed twice for 10 min with PBS and then incubated for 2 h with a goat anti-rabbit IgG antibody conjugated to Cy3 (1∶2000 dilution in PBS, Jackson ImmunoResearch, 111-165-003). The whole mounts were rinsed in PBS twice for 15 min and incubated with rhodamine–phalloidin (stock solution of 200 U/ml methanol, diluted 1∶100 in PBS for working solution) for one hour. Whole mounts were then rinsed with PBS, further microdissected into individual turns for surface preparation, and then exposed for 15 min at room temperature to the fluorescent dye 4,6-diamidino-2-phenylindole (DAPI, Sigma-Aldrich; 1.5 µg/ml in PBS) to mark nuclei. The cochlear whole mounts were rinsed in PBS and mounted on glass slides in anti-fade FluorSave reagent (Calbiochem, 34589). Hair cells in the organ of Corti were visualized by epifluorescence.

### Micro-computed Tomography (µCT)

The temporal bones and ossicles of WT and ColI(2.3)^+^/Rs1^+^12-week mice were imaged *in situ* by micro-computed tomography (µCT-50; Scanco Medical AG, Bassersdorf, Switzerland) as described [35]. The scanning region was determined by the anterior end of the sphenoid bone and extended through the posterior region of the occipital bone. The desired region was scanned at a voxel size of 4.8 µm, using an energy potential of 55 kVp and intensity of 109 µA. The region of interest was then analyzed using µCT evaluation software (version 6.0; Scanco Medical AG). The evaluation script was set at Gaussian sigma of 0.8, support of two, and lower threshold of 280 grayscale units, which corresponds to a bone density of 490 mg HA/cm^3^. Three-dimensional renderings were created using µCT 3D visualization software (version 3.8; Scanco Medical AG). False coloring of the ossicles was added using Adobe Photoshop CS.

### RNA Extraction and Quantitative Reverse Transcriptase PCR (qRT-PCR)

RNA was isolated from whole cochleae from 12-week WT and ColI(2.3)^+^/Rs1^+^ mice. Tissues were frozen in liquid nitrogen and crushed with a mortar and pestle in TRIZOL RNA isolation reagent (Invitrogen). Chloroform extraction was performed and samples were prepared for RNA purification and on-column DNase I digestion using a Qiagen RNeasy column, following the manufacturer’s instructions. RNA was reverse transcribed into cDNA using Superscript (Biorad) as recommended by the manufacturer. Quantitative PCR was performed using a ViiA 7 Real time PCR System (Life Technologies). Gene expression was assessed using Taqman probes (Life Technologies) for the Rs1 transgene (Human HTR4, Hs00168380_m1); *Mmp-13* (Mm00439491); Sclerostin (*Sost*) (Mm00470479); and *Rankl* (Mm00441906). All expression values were normalized to *Gapdh* (Mm 99999915_g1). All graphs represent fold changes of ColI(2.3)^+^/Rs1^+^ mice relative to wild type control littermates. Statistical analysis was performed using Student’s t-test with p<0.05 considered statistically significant.

## Results

### Cochlear Bone Overgrowth and Conductive Hearing Loss is Evident in a Mouse Model with Fibrous Dysplasia-like Bone Formation

**Figure 1:**
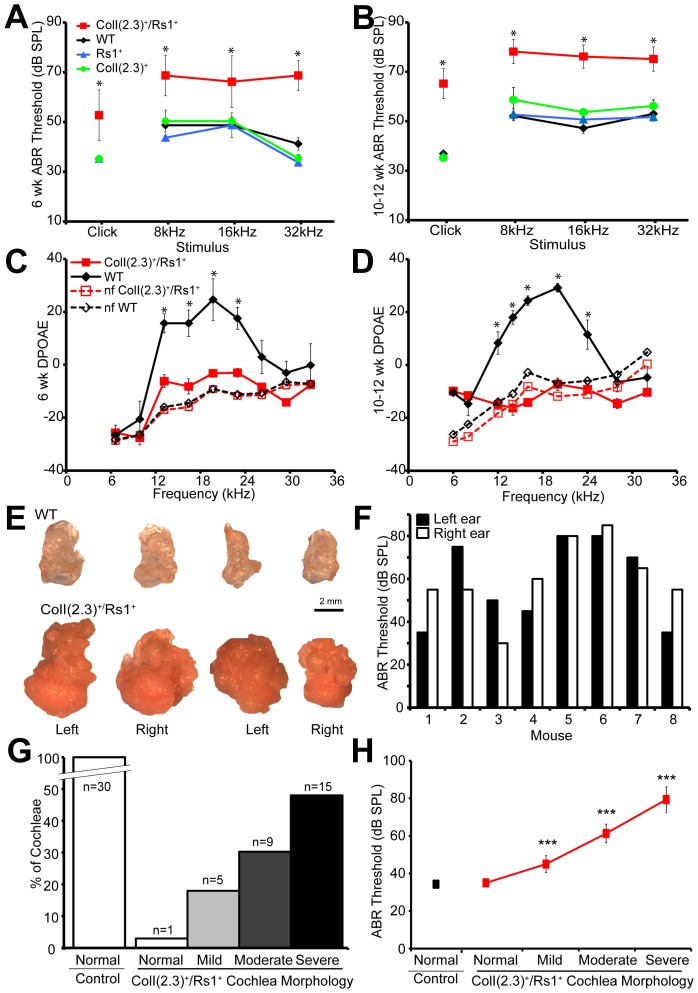
Progressive hearing loss observed in ColI(2.3)^+^/Rs1^+^ mice with fibrous dysplasia-like lesions correlates to the severity of dysplastic cochlear lesions. (**A, B**) ABR thresholds (decibels of sound pressure level, dBSPL) were measured in 6-week-old and 10–12-week-old ColI(2.3)^+^/Rs1^+^ (mutant), WT, and ColI(2.3)^−/^Rs1^+^ and ColI(2.3)^+^/Rs1^−^ (single transgenic) mice. ColI(2.3)^+^/Rs1^+^ mice showed higher ABR thresholds at. Click stimulus and at 8, 16, and 32 kHz stimuli when compared to WT and single transgenic mice at both 6- and 10–12-week-old time points. Male and female mice were analyzed together. (**C**) DPOAEs were also measured at 6-weeks and 10–12-weeks. WT and ColI(2.3)^+^/Rs1^+^ mice and compared to WT and ColI(2.3)^+^/Rs1^+^ background noise floor (nf) measurements of all experimental noise sources used. ColI(2.3)^+^/Rs1^+^ mutant DPOAEs at 6, 12, 18, 24, 32 kHz frequencies showed levels similar to nf controls compared to normal WT DPOAEs at 6 weeks of age. Male and female mice were analyzed together. (**D**) Increased differences between WT and ColI(2.3)^+^/Rs1^+^ DPOAE recordings were observed in 10–12 week-old mice. *, p<0.05. (**E**) Images of dissected cochleae from two representative sets of ColI(2.3)^+^/Rs1^+^ and WT 12-week-old mice showed gross abnormalities caused by fibrous lesion growth compared to WT cochlea. Scale bar = 2 mm. (**F**) Click ABR thresholds were measured in the left and right ears of 12-week mice (n = 8), with highly variable differences observed in the same mouse. Male and female mice were analyzed together. (**G**) A rank-order histological grading system (see methods for criteria) was used to group the severity of ColI(2.3)^+^/Rs1^+^ cochlear lesions into normal, mild, moderate, and severe lesions from 30 ColI(2.3)^+^/Rs1^+^ cochleae. When compared to 30 normal WT cochleae which all had normal morphology, chi-squared analysis showed p<0.0001. (**H**) The categorized lesions from 15 control (30 cochleae) and 15 ColI(2.3)^+^/Rs1^+^ mice (30 cochleae) were compared to measured ABR thresholds. N = 1 cochlea that appeared normal; 5 cochleae with mild lesions; 9 cochleae with moderate lesions; and 15 cochlea with severe lesions, as in (G). Male and female mice were analyzed together. ***, p<0.0001 when compared to control ABR threshold.

As previously described, ColI(2.3)^+^/Rs1^+^ mice show severe trabecular overgrowth in all bones, including those of the skull [26]. As expected, the ABR threshold of the single-transgenic mice did not differ from WT littermates at 6 or 10–12 weeks of age (Figure 1A,B). However, ColI(2.3)^+^/Rs1^+^ mice consistently showed significant ABR threshold elevations in response to click- and frequency-specific tone-burst stimuli (8, 16, and 32 kHz) (Figure 1A,B). This increase in ABR threshold was higher in the 10–12-week-old ColI(2.3)^+^/Rs1^+^ mice than in 6-week-old mice, demonstrating a progressive decline in hearing as the mice aged over this timeframe.

We next performed distortion product otoacoustic emission (DPOAE) testing to assess outer hair cell and efferent auditory function since reduced DPOAE levels can indicate outer hair cell (OHC) contractility defects or the presence of a conductive hearing deficit. Although the DPOAE responses were intact in the control mice, the DPOAE responses in ColI(2.3)^+^/Rs1^+^ mice were significantly reduced to a level close to or below the background noise level (Figure 1C,D). This loss of DPOAE in ColI(2.3)^+^/Rs1^+^ mice is thus due to either the presence of a conductive hearing loss or abnormal outer hair cell motility.

Morphological examination of the cochleae showed that most, but not all, of the cochleae from the ColI(2.3)^+^/Rs1^+^ mice had multiple spongy, bony overgrowths involving the bulla (not shown), cochlear apex, and labyrinth (Figure 1E). The phenotypic variability of the lesions was reflected in the greater standard deviations of ABRs in ColI(2.3)^+^/Rs1^+^ mice (Figure 1A), both between individual mice and between ears in the same ColI(2.3)^+^/Rs1^+^ mouse (Figure 1F). These lesions often obscured many of the traditional landmarks including the oval and round window niche. In some cases, the ossicles also appeared to be enmeshed in the bony lesions though the ossicles themselves appeared to have normal morphology. To quantify this change, we applied rank-ordered morphology scoring (Figure 1G) to the cochlea. We found that the degree of bone lesion severity correlated with the amount of hearing loss (Figure 1H), suggesting that the bony overgrowth contributed to the hearing loss observed in the ColI(2.3)^+^/Rs1^+^ mice.

Taken together, these data suggest that the hearing loss in ColI(2.3)^+^/Rs1^+^ mice was potentially conductive, possibly due to ossicular fixation. Furthermore, the variability seen in the bony overgrowth likely accounted for the variability in the ABR and DPOAE values, even between left and right cochlea in the same mouse.

### Fibrodysplastic-like Lesions Result in Thicker Otic Capsule Wall and Involve the Apex and the Labyrinthe Area of the Cochlea

**Figure 2:**
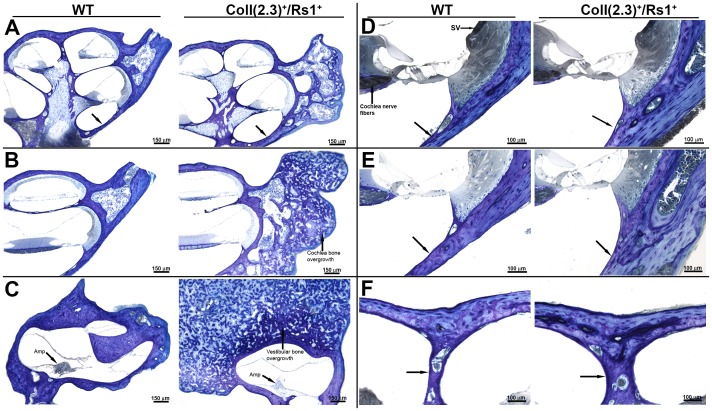
Irregular lesions in ColI(2.3)^+^/Rs1^+^ cochlea involve the apex and the labyrinth causing thickening of the otic capsule. (**A–C**) Cochlea from 12-week-old WT and ColI(2.3)^+^/Rs1^+^ mice were stained for toluidine blue and examined histologically. Mixed boney fibrous lesions were often observed surrounding ColI(2.3)^+^/Rs1^+^ cochlea compared to WT controls. ColI(2.3)^+^/Rs1^+^ cochlea had multiple fibrous boney overgrowths of the vestibular bone compared to normal WT morphology. Scale bar, 150 µm. (**D–F**) The walls of ColI(2.3)^+^/Rs1^+^ cochleae showed significant thickening in comparison to WT cochleae, possibly due to the overall thickening of the surrounding otic capsule. The stria vascularis (SV) in the ColI(2.3)^+^/Rs1^+^ mice appear normal. Scale bar, 100 µm.

We next used detailed histological analysis to better understand the microscopic changes occurring within the cochlea in ColI(2.3)^+^/Rs1^+^ mice. The ColI(2.3)^+^/Rs1^+^ mice showed aggressive bony and fibrous overgrowths that surrounded the otic capsule and the adjacent vestibular labyrinth as compared to WT controls (Figure 2A–C). The most severely affected areas were in bone adjacent to the cochlea and the labyrinth. Although the FD lesions often increased the thickness of the outer wall of the cochlea (Figure 2A, 2D–E, black arrows) as well as the mid-modiolar bone separating the apical scala (Fig. 2F black arrows), no lesions were observed involving the inner cortex of the otic capsule or centrally in the bony spiral modiolus (Figure 2A). The stria vascularis (SV), organ of Corti (OC) and tunnel of Corti (TC) (Figure 2A–B, 2D–E) were also normal. Thus, although the otic capsule bone showed some histologic defects, whether the hearing loss in ColI(2.3)^+^/Rs1^+^ mice is sensorineural or conductive remained unclear.

### Intact Sensorineural Structures of ColI(2.3)^+^/Rs1^+^ Cochlea

**Figure 3:**
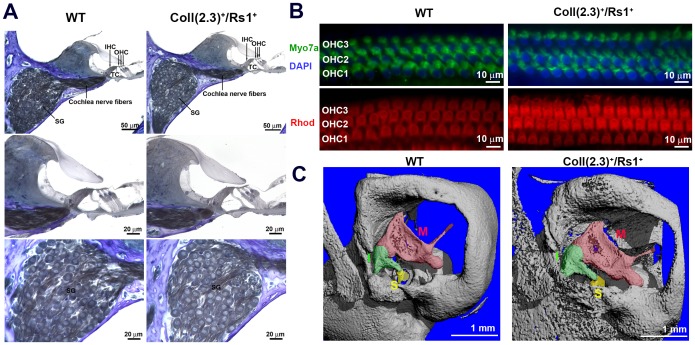
Sensorineural structures of ColI(2.3)^+^/Rs1^+^ mice are normal despite the bony overgrowth affecting the ossicular chain. (**A**) Histological analysis of the sensorineural structures of 12-week-old WT and ColI(2.3)^+^/Rs1^+^ revealed no gross abnormalities in the organ of Corti (OC), tunnel of Corti (TC), inner hair cells (IHC), or outer hair cells (OHC). (**B**) Whole mount immunofluorescence preparations of the outer hair cells revealed no visible differences in hair cell structure or number in ColI(2.3)^+^/Rs1^+^ in comparison to WT structures. (**C**) Micro computed x-ray tomography of the temporal bones was examined. A region of interest including the middle ear was selected in WT and ColI(2.3)^+^/Rs1^+^ at 12-week-old mice. ColI(2.3)^+^/Rs1^+^ temporal bones showed significant bony overgrowth of the middle ear compared to WT. The ossicles are structurally identifiable and are affected in the ColI(2.3)^+^/Rs1^+^ mice [malleus (M), incus (I), and stapes (S)].

We next examined the integrity of the organ of Corti, spiral ganglion (SG), inner hair cells (IHCs), outer hair cells (OHCs), and cochlear nerve fibers to assess if the observed hearing loss could be caused by sensory or neuronal defects (Figure 3A). Cells in the organ of Corti and spiral ganglia had normal size and morphology. Cochlear mid-turn surface preparations stained with Myosin 7a, a marker for hair cells [32], [33], demonstrated normal numbers of OHCs. Additional labeling with rhodamine, a stain for actin, also revealed that OHC steriocillia and cuticular plates were present in both the WT and ColI(2.3)^+^/Rs1^+^ cochlea (Figure 3B). These results showed no significant histological differences between WT and ColI(2.3)^+^/Rs1^+^ cochlear sensorineural structures.

To more fully examine the integrity of bony structures in the ear, we performed microCT analysis of the temporal bones. In some cochlea, the ossicles were directly affected with malformations and increased bone, as well as impingement by FD lesions originating from surrounding bone (Figure 3C). In contrast, WT cochlea showed normal morphology.

These morphometric and histologic analyses demonstrate that the observed hearing loss in the ColI(2.3)^+^/Rs1^+^ mice was predominantly due to conductive loss from the FD lesions impinging upon the ossicular chain and sound conduction mechanism, with no objective evidence of a sensory or neural component to the hearing loss.

### Defective Regulation of Bone Remodeling in ColI(2.3)^+^/Rs1^+^ Cochlea

We next sought to understand the molecular mechanisms that led to the striking bony overgrowth in the cochlea in the ColI(2.3)^+^/Rs1^+^ mice. In long bones of ColI(2.3)^+^/Rs1^+^ mice, high levels of G_s_ activation resulted in significantly increased bone remodeling and increased markers of bone turnover [26]. However, normal cochlear bone does not undergo classical osteoblast- and osteoclast-mediated remodeling [3], making it unclear what cellular mechanisms induce temporal bone fibrous dysplasia formation.

**Figure 4:**
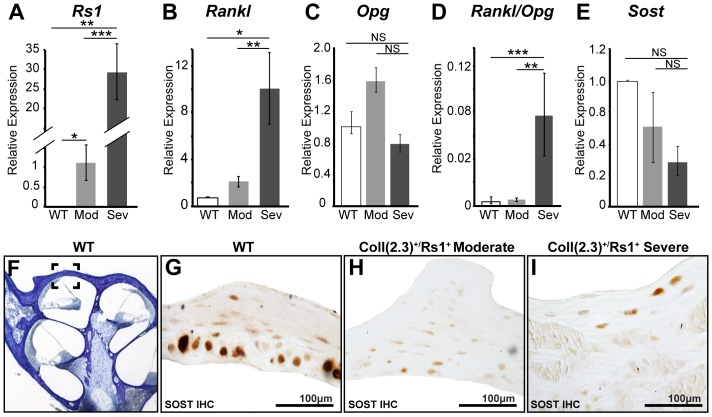
Markers of osteoclast-mediated bone remodeling is reactivated in ColI(2.3)^+^/Rs1^+^ cochlea. Markers of bone remodeling were assessed by immunohistochemistry on dissected 12-week-old male WT and ColI(2.3)^+^/Rs1^+^ cochlea with moderate (Mod) and severe (Sev) fibrous lesions using quantitative PCR. (**A**) Rs1 transgene expression was absent in WT controls and elevated in moderate (**, p = 0.01) and elevated 30-fold in severe ColI(2.3)^+^/Rs1^+^ cochlea (***, p<0.001). (**B**) *Rankl*, a marker for osteoclast differentiation and activity was significantly increased in ColI(2.3)^+^/Rs1^+^ severe cochlea when compared to WT and moderate ColI(2.3)^+^/Rs1^+^ cochlea (*, p<0.05; **, p<0.01). No statistically significant differences were observed in moderate ColI(2.3)^+^/Rs1^+^ cochlea compared to WT (p = 0.63). (**C**) There were also no statistically significant changes observed in *Osteoprotegrin* expression in moderate and severe ColI(2.3)^ +^/Rs1^+^ cochlea compared to WT cochlea (p = 0.29, and p = 0.645 respectively). (**D**) The ratios of *Rankl* and osteoclast inhibitory factor *osteoprotegrin* (*Opg*) were significantly increased in ColI(2.3)^+^/Rs1^+^ severe cochlea compared to moderate ColI(2.3)^+^/Rs1^+^ (**, p<0.01) and WT cochlea (***, p<0.005) indicating that osteoclast mediated remodeling is increased in ColI(2.3)^+^/Rs1^+^ severe cochlea. (**E**) No statistically significant changes were observed in *Sost* expression in ColI(2.3)^+^/Rs1^+^ moderate and severe cochlea compared to WT expression (p = 0.73 and p = 0.43 respectively). Error bars are mean +/− SEM of triplicate measurements, n = 5 WT and 5 each of ColI(2.3)^+^/Rs1^+^ moderate and severe cochleae. (**F**) A specific area of the otic capsule (indicated by box) was examined immunohistochemical analysis. (**G–I**) Immunohistochemistry for SOST showed visibly significant decreases in expression in moderate and severe ColI(2.3)^+^/Rs1^+^ cochlea compared to WT cochlea.

**Figure 5:**
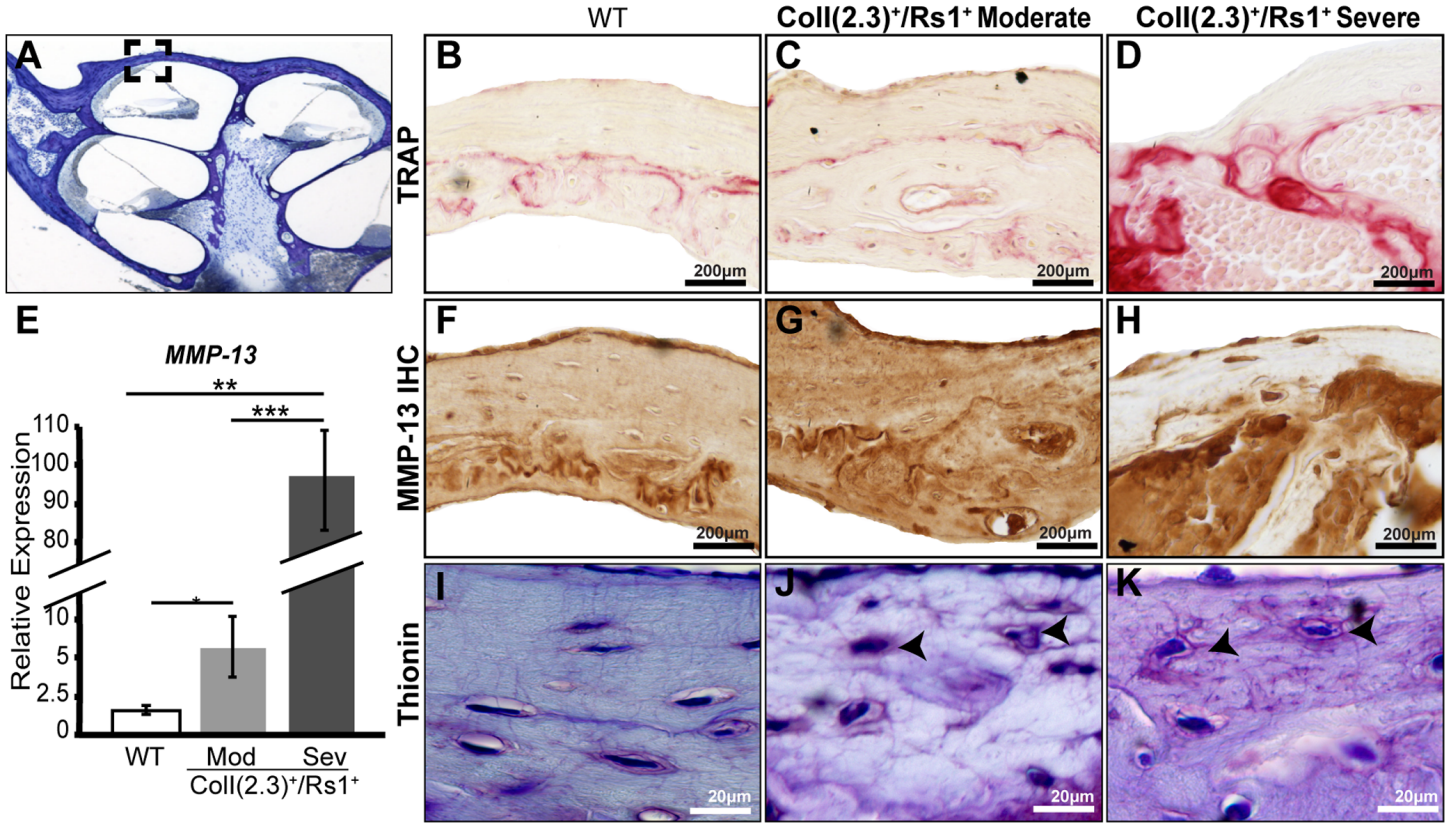
Abnormal peri-lacunar remodeling in cochlear fibrous dysplasia. (**A**) 12-week-old cochlea from male WT and ColI(2.3)^+^/Rs1^+^ cochleae were examined for markers of peri-lacunar remodeling in a specific area of the otic capsule. (**B–D**) TRAP, a marker for osteoclast activity, was elevated in moderate and severe ColI(2.3)^+^/Rs1^+^ otic capsules compared to the low levels observed in WT controls. (**E**) The expression of osteocyte secreted remodeling factor, MMP-13 was significantly increased 7-fold in moderate (**, p<0.005) and 100 fold in severe ColI(2.3)^+^/Rs1^+^ cochleae (***, p<1×10^−05^) compared to WT cochleae. (n = 6 male WT, n = 5 male ColI(2.3)^+^/Rs1^+^ moderate, and n = 5 male ColI(2.3)^+^/Rs1^+^ severe cochlea). Error bars are mean +/− SEM of triplicate measurements. (**F–H**) Immunohistochemistry showed that MMP-13 expression was confined to the otic capsule in WT cochleae, but the domain of expression is increased in ColI(2.3)^+^/Rs1^+^ moderate and severe cochlea. (**I–K**) Thionin staining of the canalicular network was examined in WT and ColI(2.3)^+^/Rs1^+^ moderate and severe cochleae. The canalicular network in WT cochlea shows normal elliptical osteocyte morphology and connectivity. ColI(2.3)^+^/Rs1^+^ moderate and severe cochleae show an abnormal rounded osteocyte morphologies with disrupted and disorganized canalicular networks of varying severity (n = 5 males per genotype).

To determine if variable expression of key bone cell regulatory factors were responsible for severity of the FD lesions seen in the transgenic mice, we compared the gene expression in the WT cochleae *vs*. moderate and severely affected ColI(2.3)^+^/Rs1^+^ cochleae. As expected, Rs1 expression in the total cochleae was higher in the severely affected samples (Figure 4A). *Rankl*, a secreted factor produced by osteoblasts and osteocytes that increases osteoclast recruitment, differentiation, activity, and survival [36], showed significantly higher expression in the ColI(2.3)^+^/Rs1^+^ cochlea, and was more dramatically elevated in severe ColI(2.3)^+^/Rs1^+^ lesions surrounding the cochlea (Figures 4B and 5D). Interestingly, no changes were observed in the mRNA levels of the osteoblast secreted RANKL antagonist, *osteoprotegrin* (*Opg*), in moderate and severe ColI(2.3)^+^/Rs1^+^ cochlea compared to WT (Figure 4C). *Rankl*/*Opg* ratios [37] used as a measure of osteolytic balance [38] were significantly increased in the severely-affected ColI(2.3)^+^/Rs1^+^ cochleae as compared to ColI(2.3)^+^/Rs1^+^ moderate and WT cochlea (Figure 4D).

We next examined the expression of sclerostin, a protein encoded by the gene *Sost,* to assess osteocyte activity. Sclerostin is an osteocyte-derived antagonist of the osteoinductive Wnt signaling pathway [39], [40]. Normally, Sclerostin suppresses new bone formation [40]. Loss-of-function mutations in sclerostin result in progressive bone overgrowth in diseases, such as sclerosteosis [41] and van Buchem’s disease [42], which are also associated with hearing loss. To determine if this regulatory mechanism was also impaired in the FD-like lesions in the cochlea of ColI(2.3)^+^/Rs1^+^ mice, we assessed sclerostin protein and *Sost* gene expression by immunohistochemistry and quantitative PCR. We observed no statistically significant decreases in *Sost* mRNA expression in moderate or severe ColI(2.3)^+^/Rs1^+^ cochlea compared to WT cochlea (Figure 4E). However, immunohistochemistry revealed that Sclerostin protein expression was qualitatively decreased in moderate and severe ColI(2.3)^+^/Rs1^+^ lesions compared to WT (Figure 4F–I). Taken together, these data suggest that ColI(2.3)^+^/Rs1^+^ cochlea may exhibit increased bone remodeling due in part to decreased sclerostin expression by osteocytes, which may activate osteoblast mediated bone remodeling [43].

### Perilacunar Remodeling is Disrupted in FD Cochlea

Perilacunar remodeling (PLR) is mediated by osteocytes in the canalicular network that remodel the local bone matrix to maintain bone quality and systemic mineral homeostasis [40], [44]–[47]. In PLR, osteocytes, in addition to osteoclasts, secrete tartrate resistant acid phosphatase (TRAP) [47] and matrix metalloproteinases (MMPs) [34], [48], [49] to drive bone resorption and remodeling.

We thus focused on *Rankl* and *MMP-13* to investigate the role of PLR in normal and ColI(2.3)^+^/Rs1^+^ cochlear bone. *Rankl* mRNA expression was elevated in the ColI(2.3)^+^/Rs1^+^ cochlear bone (Figure 4B), as was TRAP staining of histological sections (Figures 5A–D). We found that *Mmp-13* mRNA expression was low in WT cochleae; in contrast, *Mmp-13* mRNA expression was significantly increased in both moderate and severe double mutant cochleae (Figure 5E). In the WT cochlea, MMP-13 protein was highly localized within the otic capsule (Figure 5F), in the same areas where Sclerostin and TRAP are maximally expressed (Figures 4G and 5B). In the ColI(2.3)^+^/Rs1^+^ cochlea not only are *Mmp-13* mRNA and protein levels increased (Figure 5E), but the normal distinctive localization is also disrupted (Figure 5G, H) with increased expression in both osteocytes and in cochlear bone matrix. Thus, in the severe FD-like lesions, the level and localization of TRAP and MMP-13 expression is greatly expanded beyond the narrow zones in which they are normally expressed (Figures 5D, H and 5B, F, respectively).

Integrity of the canalicular network is normally required to maintain osteocyte viability and function, which directly influences bone quality [50] as demonstrated in MMP-13 deficient mice, which show defective PLR and a disrupted canalicular network [34]. To identify if a similar disruption occurs in the ColI(2.3)^+^/Rs1^+^ mice, we used thionin staining to visualize the canalicular network of 12-week moderately and severely affected ColI(2.3)^+^/Rs1^+^ cochleae. The thionin staining revealed highly disorganized dendritic processes and abnormal osteocyte morphology relative to the aligned canalicular organization observed in WT cochlear bone (Figure 5J, K, and I respectively).

Taken together, these results demonstrate that the normally limited bone remodeling and turnover in the cochlea is hyperactivated in moderate and severe ColI(2.3)^+^/Rs1^+^ cochleae, and is accompanied by decreased levels of sclerostin and increased markers of osteoclast and perilacunar bone remodeling (TRAP, RANKL, and MMP-13).

## Discussion

Many fibrous dyplasia patients develop progressive hearing loss through mechanisms that remain unclear. In this study, we investigated the cause of fibrous dysplasia-associated hearing loss using the ColI(2.3)^+^/Rs1^+^ mouse model that develops invasive FD-like lesions [26]. We found that ColI(2.3)^+^/Rs1^+^ mice developed progressive hearing loss due to FD-like lesions that often surrounded the ossicular chain and obstructed the oval and round window regions of the cochlea. These bony defects prohibited sound propagation into the cochlea, resulting in elevations in ABR and loss of DPOAEs. Further, the elevation of ABR thresholds significantly correlated with the severity of FD lesions in the cochlea. Histological and immunofluorescent analysis of the organ of Corti revealed no abnormalities, decreasing the likelihood that a sensorineural deficit caused the hearing loss.

We found that the severity of hearing loss and bony lesions in the ColI(2.3)^+^/Rs1^+^ mice correlated with deregulation of factors implicated in the control of bone remodeling, including RANKL, SOST, TRAP, and MMP-13. Relative to WT littermates, ColI(2.3)^+^/Rs1^+^ cochleae had lower levels of factors that suppress bone remodeling, but higher levels of factors that promote it. Specifically, we found that the expression of SOST, an osteocyte-specific negative regulator of bone remodeling [40], was significantly reduced in the ColI(2.3)^+^/Rs1^+^ cochleae and correlated to the severity of graded FD lesions. In contrast, TRAP, a marker for active bone remodeling by osteoclasts and osteocytes [47], was increased in moderate and severely affected ColI(2.3)^+^/Rs1^+^ cochleae. This area of TRAP overexpression was most pronounced in the otic capsule.

We and other groups previously showed that PLR is essential for maintaining the osteocyte canalicular network [34], [47]. MMP-13 is a key PLR enzyme, and its deficiency causes disruption of the canalicular network [34]. Here, we observed increased MMP-13 expression in moderate and severe ColI(2.3)^+^/Rs1^+^ cochlear lesions compared to WT cochleae. Elevated MMP-13 expression was also associated with canalicular disorganization, often with enlarged canalicular channels around osteocytes and with altered morphology. In addition, we consistently observed MMP-13 staining in the pericellular matrix and intracellular space, in addition to the bone matrix staining that is typically observed. This altered MMP-13 localization in the severe FD mice may indicate that Rs1 expression is associated with a protein processing defect or change in the localization of bone matrix proteins, and may account for a decrease in bone matrix maturation we observed previously in long bones [51]. Collectively, these results suggest that elevated G_s_ signaling in ColI(2.3)^+^/Rs1^+^ mice, and possibly also in FD patients, may cause hyper-activation of osteocyte-mediated PLR as well as bone remodeling by osteoclasts and osteoblasts, ultimately leading to hearing loss by bony overgrowths that affect the conductive mechanisms of the ear.

Surprisingly, while the FD-like lesions were easily identified in the bony structures surrounding the cochlea, the most severe lesions largely spared the cochlea itself. We speculate that this protection may result from the unique regulation of bone remodeling in the cochlea – which is very limited relative to bone remodeling in the rest of the skeleton [8]. A key inhibitor of osteoclast activity, osteoprotegrin (OPG), is normally enriched in the cochlea relative to other sites in the body [52]. In mice, OPG is produced at high levels by fibrocytes within the spiral ligament and secreted in the perilymph [52], [53]. Mice deficient in OPG show excessive remodeling throughout the middle and inner ear resulting in severe hearing loss [3]. Similar pathology is also found in human patients with Juvenile Paget’s disease [54]. We found that the high level of *Opg* expression in the cochlea was maintained in the ColI(2.3)^+^/Rs1^+^ cochleae. Therefore, even though osteoblastic-cell-specific activation of G_s_-GPCR signaling in our ColI(2.3)^+^/Rs1^+^ mouse model increases the *Rankl*/*Opg* ratios, the elevated cochlear OPG may be sufficient to spare cochlear bone from the most severe FD lesions. These findings in the cochlea motivate further study on the possible role for OPG in suppressing the progression of FD lesions in the ear or at other sites.

While there are cases of FD causing sensorineural hearing loss in humans, it has been hypothesized that this is due to auditory neural compression from the FD bone changes [18]. The results in our mouse model with FD like lesions would support that mechanism, as well as possibly physical changes to the ossicular chain, as opposed to pathologic changes of sensory structures as seen in such lesions as cochlear otosclerosis [10]. These results point to a role for peri-lacunar remodeling in the cochlea that may be disrupted in FD.

In conclusion, our results show that the cochlea is a unique bony structure characterized by limited bone turnover that confers protection from proliferative and metabolically active FD bony lesions. The invasive fibro-osseous lesions seen in this mouse model cause conductive hearing loss through involvement of the ossicular chain, in a manner very similar to that seen in humans. These mechanisms could be new pharmacologic targets to treat the skeletal or hearing manifestations of FD or other skeletal diseases.
